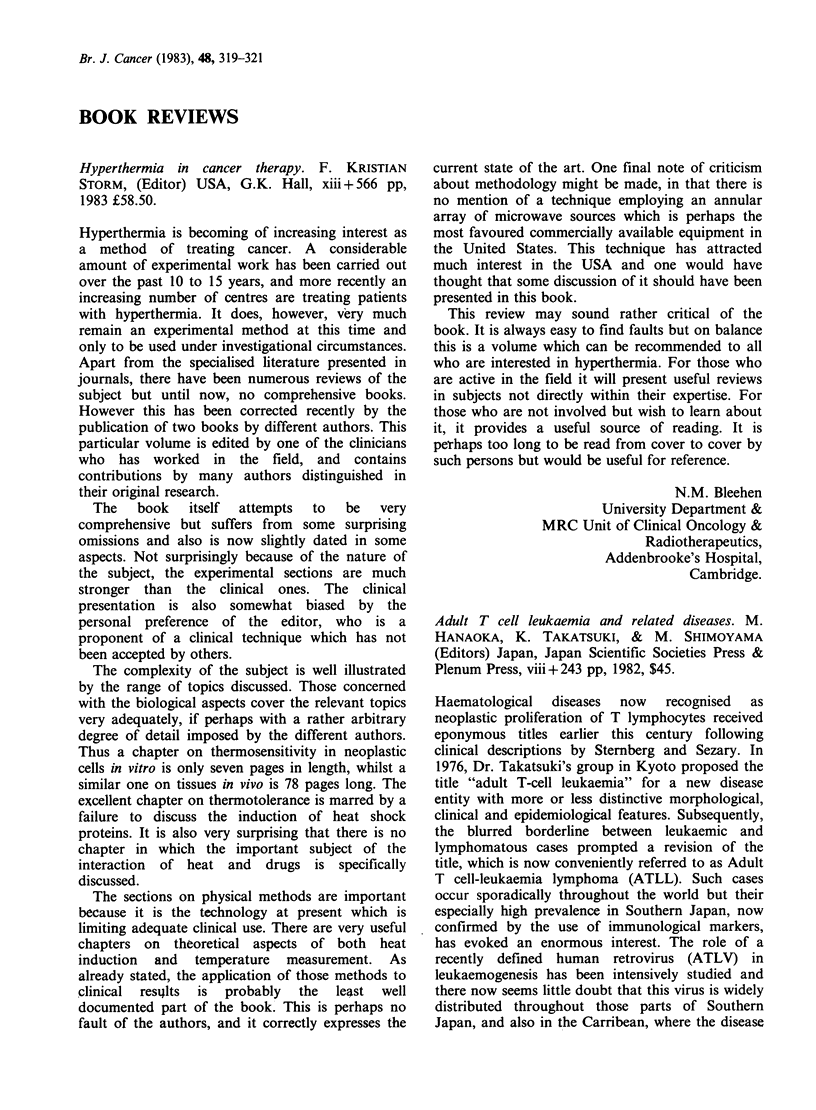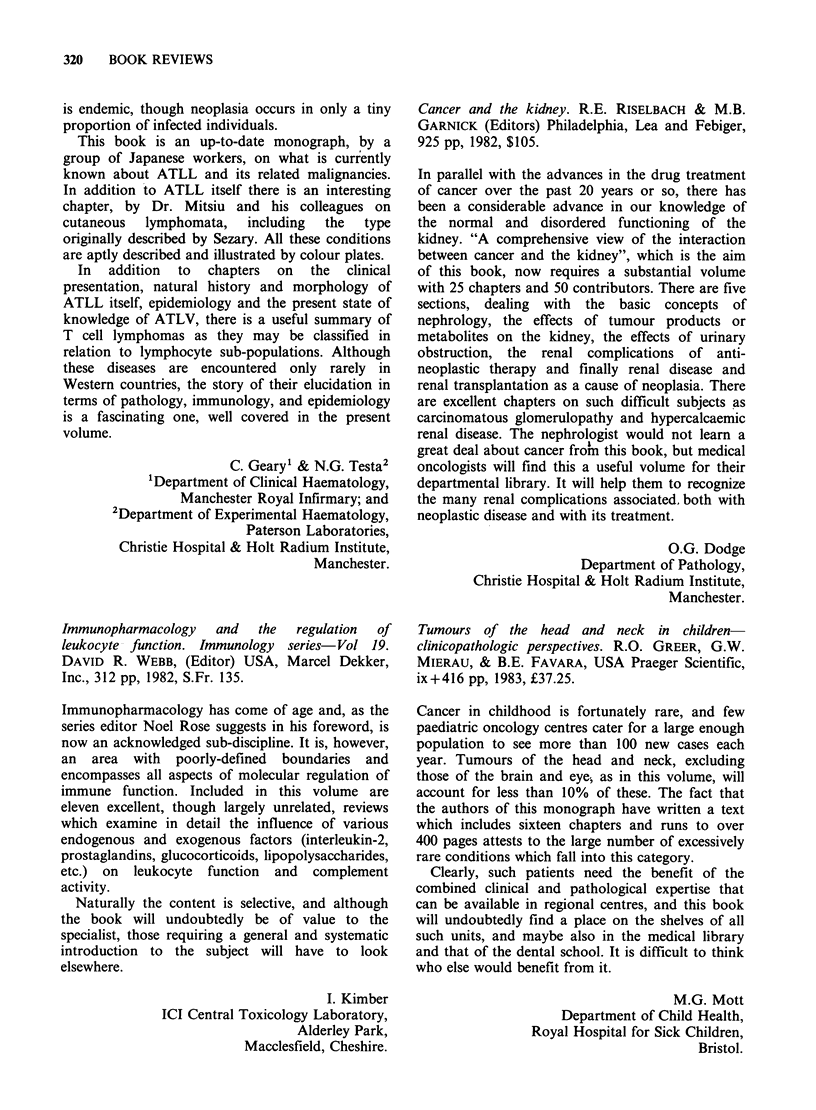# Adult T cell leukaemia and related diseases

**Published:** 1983-08

**Authors:** C. Geary, N.G. Testa


*Adult T cell leukaemia and related diseases*. M. HANAOKA, K. TAKATSUKI, & M. SHIMOYAMA (Editors) Japan, Japan Scientific Societies Press & Plenum Press, viii+243 pp, 1982, $45.

Haematological diseases now recognised as neoplastic proliferation of T lymphocytes received eponymous titles earlier this century following clinical descriptions by Sternberg and Sezary. In 1976, Dr. Takatsuki's group in Kyoto proposed the title "adult T-cell leukaemia" for a new disease entity with more or less distinctive morphological, clinical and epidemiological features. Subsequently, the blurred borderline between leukaemic and lymphomatous cases prompted a revision of the title, which is now conveniently referred to as Adult T cell-leukaemia lymphoma (ATLL). Such cases occur sporadically throughout the world but their especially high prevalence in Southern Japan, now confirmed by the use of immunological markers, has evoked an enormous interest. The role of a recently defined human retrovirus (ATLV) in leukaemogenesis has been intensively studied and there now seems little doubt that this virus is widely distributed throughout those parts of Southern Japan, and also in the Carribean, where the disease is endemic, though neoplasia occurs in only a tiny proportion of infected individuals.

This book is an up-to-date monograph, by a group of Japanese workers, on what is currently known about ATLL and its related malignancies. In addition to ATLL itself there is an interesting chapter, by Dr. Mitsiu and his colleagues on cutaneous lymphomata, including the type originally described by Sezary. All these conditions are aptly described and illustrated by colour plates.

In addition to chapters on the clinical presentation, natural history and morphology of ATLL itself, epidemiology and the present state of knowledge of ATLV, there is a useful summary of T cell lymphomas as they may be classified in relation to lymphocyte sub-populations. Although these diseases are encountered only rarely in Western countries, the story of their elucidation in terms of pathology, immunology, and epidemiology is a fascinating one, well covered in the present volume.

C. Geary[1] & N.G. Testa[2]
[1]Department of Clinical Haematology,
Manchester Royal Infirmary; and
[2]Department of Experimental Haematology,
Paterson Laboratories,
Christie Hospital & Holt Radium Institute,
Manchester.

Christie Hospital & Holt Radium Institute,

Manchester.